# Combination of endobronchial bronchoscopic debulking and bronchoplastic segmentectomy of an obstructive neuroendocrine tumour: probably the least invasive approach

**DOI:** 10.1093/icvts/ivac032

**Published:** 2022-02-25

**Authors:** Vladimir Mégevand, Jon A Lutz, Gregor J Kocher, Philippe Dumont

**Affiliations:** 1 Department of Surgery, Hôpitaux Universitaires de Genève, Geneva, Switzerland; 2 Thoracic Unit, Department of Surgery, Hôpital Cantonal de Fribourg, Fribourg, Switzerland; 3 Division of General Thoracic Surgery, Inselspital Bern, Bern University Hospital, Bern, Switzerland; 4 Department of Internal Medicine and Pneumology, Hôpital Cantonal de Fribourg, Fribourg, Switzerland

**Keywords:** Neuroendocrine tumour, Endoscopic debulking, Uniportal segmentectomy, Bronchoplasty

## Abstract

We report the case of a female patient with an obstructing well-differentiated neuroendocrine tumour in the apical segment of the completely atelectatic right lower lobe. Bronchoscopic debulking of the tumour lead to re-ventilation of the remaining lobe, allowing to perform a lung-sparing bronchoplastic resection of the affected segment by uniportal video-assisted thoracic surgery.

## INTRODUCTION

Neuroendocrine tumours (NETs) represent <3% of all primary lung neoplasms and treatment options have evolved in recent years [1]. Although the gold standard is complete surgical resection with lymphadenectomy [2], minimally invasive and lung-sparing techniques in the form of sublobar anatomical resections are gradually replacing the classical lobectomy by thoracotomy approach [3]. In the past 25 years, the evolution of bronchoscopic therapy—especially for centrally located intraluminal NETs—has challenged surgery as an initial therapy [4–6]. In this case, we show how combining the latest advances in interventional bronchoscopy and uniportal video-assisted thoracic surgery enabled us to resect a well-differentiated NET (grade 1) originating in the apical segment of a completely atelectatic lower lobe.

## CASE REPORT

We report the case of a 43 years old, otherwise healthy caucasian female patient, who presented with a chronic irritative cough and episodes of recurrent productive bronchitis for 24 months without intercurrent resolution. No history of shortness of breath, asthma, chest pain, weight loss or fever was reported. The clinical examination was normal except for fluctuating ronchi located at the right pulmonary base.

**Figure 1:**
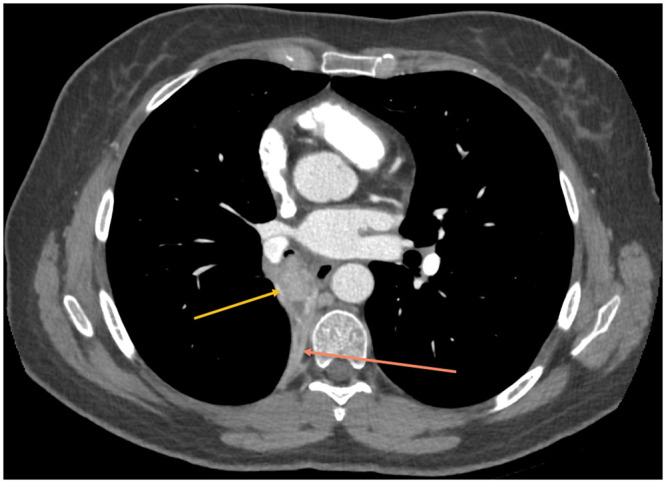
Thoracic computed tomography scan revealing obstruction of the right lower bronchus by a contrast-enhanced intraluminal lesion of 2.5 cm in diameter.

Pulmonary function tests were normal with an FEV1 of 2.66 l/s (102%) and a CO-diffusion capacity of 89%, especially no bronchial hyperreactivity to metacholine was noticed. Thoracic computed tomography scan (Fig. 1) revealed a complete atelectasis of the right lower lobe with obstruction of the right lower bronchus by a contrast-enhanced intraluminal lesion of 2.5 cm in diameter and some slightly enlarged hilar lymph nodes. The urine dosage of 5-HIAA was not elevated.

Bronchoscopy was performed and a budding tumour originating from the apical segment (S6) of the right lower lobe was found. The lesion was resected with a diathermic loop in combination with a cryoprobe and sent for histopathological analysis (Video 1). Histology revealed a grade 1 NET and decision was taken for resection at the thoracic oncology board.

**Figure 2:**
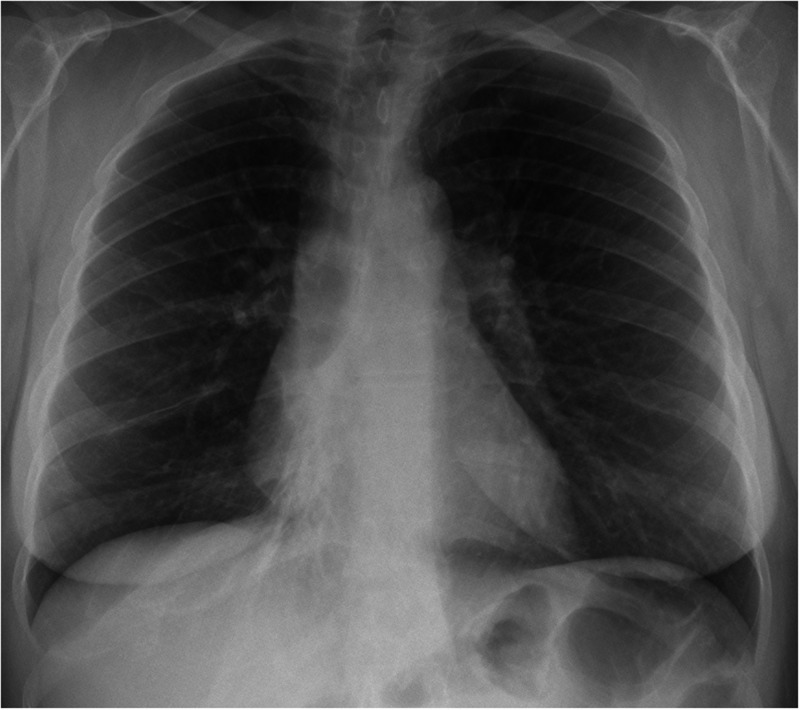
Chest X-ray at postoperative Day 3 showing a well-expanded right lung with only minor residual atelectasis at the base.

A uniportal thoracoscopic bronchoplastic S6-segmentectomy of the right lower lobe was performed with radical mediastinal lymphadenectomy. The B6-bronchus was resected V-shaped under bronchoscopic control and the margins were tumour-free at fresh frozen section. Bronchoplasty was performed with interrupted stitches of 3.0 PDS. At the end of the operation, the remaining right lower lobe was well ventilated and the perfusion was complete, confirmed by ICG-fluorescence. The patient was discharged on postoperative Day 3 after safe removal of the chest tube. Subsequent chest X-ray (Fig. 2) showed a well-expanded right lung with only minor residual atelectasis at the base.

Computed tomography scan at 1 year follow-up (Video 2) showed no signs of recurrence and a well-ventilated basal pyramid.

## DISCUSSION

Approximately 20–30% of NETs affect the lung, which is the second most common location for NETs after the gastrointestinal tract [1, 2]. Well-differentiated NETs of the lung (low-grade NETs) have a higher potential for resectability and a better prognosis than higher grade NETs [2]. They usually origin in the central airways and typically produce symptoms and signs associated with airway obstruction such as cough, wheezing or haemoptysis [2, 3]. Chest X-ray usually shows nodular lesions with lobulated edges. If parenchymal alterations are observed as in our case, atelectasis or obstructive pneumonia may be associated. Contrast-enhanced computed tomography specifies the radiological findings observed on the chest X-ray and usually describes well-vascularized tumours [3].

Bronchoscopy helps determining the final diagnosis in the majority of cases. Low-grade NETs have a reputation for being a major source of bleeding, especially during biopsies. However, very low incidence of post-interventional massive haemorrhage and no lethal complication have been reported in the literature [4]. To increase the safety of the procedure and obtain good-quality samples, some authors recommend the use of adrenaline before the biopsy [5]. Surgical resection is the treatment of choice for such tumours; however, an initial endobronchial resection may be considered for centrally located NETs [6] and necessitates completion surgery when the tumour is not radically resected or if a higher grade NET is found. Primary desobstruction is helpful in Fast Track programmes, aiding patients to recovery pulmonary function in order to perform preoperative exercises and physiotherapy.

In this case, we proceeded to an endobronchial debulking of the lesion located at the orifice of the apical segment of the right lower lobe. This procedure helped us determine the histological diagnosis and allowed a better ventilation of the remaining lower lobe; therefore, precising the tumour’s location. After the procedure, the exact origin of the tumour was clear and a minimally invasive lung-sparing R0-resection of the remaining tumour was possible. Without desobstruction of the intermediate bronchus, the existing secretions could have evolved into a pneumonia and we probably would have had no other choice but to perform minimally invasive lobectomy or open exploration with a less extensive resection.

## CONCLUSION

Initial bronchoscopic debulking has been shown to be safe and even curative in some cases of centrally located NETs. In tumours located in the segmental bronchus like in this case, the initial approach by endobronchial resection allows to reduce the extent of the surgery in terms of access and the volume of lung resection without compromising oncological radicality or the patient’s outcome.

**Conflict of interest:** none declared.

**Reviewer information:** Interactive CardioVascular and Thoracic Surgery thanks Joao-Carlos Das-Neves-Pereira and the other anonymous reviewers for their contribution to the peer review process of this article.

## References

[1] Hendifar A, Marchevsky A, Tuli R. Neuroendocrine tumors of the lung: current challenges and advances in the diagnosis and management of well-differentiated disease. J Thorac Oncol 2017;12:425–36. 27890494 10.1016/j.jtho.2016.11.2222

[2] Filosso P, Rena O, Donati G, Casadio C, Ruffini E, Papalia E et al Bronchial carcinoid tumors: surgical management and long-term outcome. J Thorac Cardiovasc Surg 2002;123:303–9. 11828290 10.1067/mtc.2002.119886

[3] Ghaly G, Kamel M, Nasar A, Paul S, Lee PC, Port J et al Video-assisted thoracoscopic surgery is a safe and effective alternative to thoracotomy for anatomical segmentectomy in patients with clinical stage I non-small cell lung cancer. Ann Thorac Surg 2016;101:465–72. 26391692 10.1016/j.athoracsur.2015.06.112

[4] Gao Y, Moua T, Midthun D, Mullon J, Decker P, Ryu J. Diagnostic yield and complications associated with bonchoscopic biopsy of endobronchial carcinoid tumors. J Bronchology Interv Pulmonol 2020;27:184–9. 31876538 10.1097/LBR.0000000000000639

[5] Sutedja T, Schreurs A, Vanderschueren R, Kwa B, Vd Werf T, Postmus P. Bronchoscopic therapy in patients with intraluminal typical carcinoid. Chest 1995;107:556–68. 7842794 10.1378/chest.107.2.556

[6] Brokx H, Risse E, Paul M, Grünberg K, Golding R, Kunst P et al Initial bronchoscopic treatment for patients with intraluminal bronchial carcinoids. J Thorac Cardiovasc Surg 2007;133:973–8. 17382637 10.1016/j.jtcvs.2006.12.013

